# Development of a copper-clioquinol formulation suitable for intravenous use

**DOI:** 10.1007/s13346-017-0455-7

**Published:** 2017-12-16

**Authors:** Moe Wehbe, Armaan K. Malhotra, Malathi Anantha, Cody Lo, Wieslawa H. Dragowska, Nancy Dos Santos, Marcel B. Bally

**Affiliations:** 1 Experimental Therapeutics, British Columbia Cancer Agency, 675 West 10th Avenue, Vancouver, BC V5Z 1L3 Canada; 2 Faculty of Pharmaceutical Sciences, University of British Columbia, 2146 East Mall, Vancouver, BC V6T 1Z3 Canada; 3 Department of Pathology and Laboratory Medicine, University of British Columbia, 2211 Wesbrook Mall, Vancouver, BC V6T 2B5 Canada; 4 Center for Drug Research and Development, Vancouver, BC V6T 1Z4 Canada

**Keywords:** Clioquinol, Copper, Cancer, Copper complexes, Liposomes, Disulfiram

## Abstract

Clioquinol (CQ) is an FDA-approved topical antifungal agent known to kill cancer cells. This facilitated the initiation of clinical trials in patients with refractory hematologic malignancies. These repurposing efforts were not successful; this was likely due to low intracellular levels of the drug owing to poor absorption and rapid metabolism upon oral administration. CQ forms a sparingly soluble copper complex (Cu(CQ)_2_) that exhibits enhanced anticancer activity in some cell lines. We have utilized a novel method to synthesize Cu(CQ)_2_ inside liposomes, an approach that maintains the complex suspended in solution and in a format suitable for intravenous administration. The complex was prepared inside 100-nm liposomes composed of 1,2-distearoyl-sn-glycero-3-phosphocholine/cholesterol (55:45). The therapeutic activity of the resultant formulation was evaluated in two subcutaneous tumor models (glioblastoma and ovarian cancers) but was not active. We also assessed the ability of the Cu(CQ)_2_ formulation to increase copper delivery to cancer cells in vitro and its potential to be used in combination with disulfiram (DSF). The results suggested that addition of Cu(CQ)_2_ enhanced cellular copper levels and the activity of DSF in vitro; however, this combination did not result in a statistically significant reduction in tumor growth in vivo. These studies demonstrate that a Cu(CQ)_2_ formulation suitable for intravenous use can be prepared, but this formulation used alone or in combination with DSF was not efficacious. The methods described are suitable for development formulations of other analogues of 8-hydroxyquinoline which could prove to be more potent.

## Introduction

Clioquinol (CQ) was commonly used as an oral antimicrobial agent for treating diarrhea and skin infections [1, 2]. However, in the 1960s, its use in Japan was associated with a debilitating neurological disorder referred to as subacute myelo-optic neuropathy (SMON). This eventually led to CQ being withdrawn from the market [1, 3]. Interestingly, epidemiologic reports suggest that CQ was not responsible for SMON and no other population showed a similar adverse response [1, 2]. Today, CQ is commonly used as a topical antibiotic under the trade name Vioform® [2] and more recently, this drug has been the focus of repurposing efforts for the treatment of Alzheimer’s disease [4, 5] and cancer [6, 7].

This study focuses on the potential use of CQ as an anticancer agent. It has been noted that the anticancer effects of CQ are enhanced when it is administered as a copper CQ (Cu(CQ)_2_) complex [6–10]. The structure of Cu(CQ)_2_ has been characterized [11]; however, the mechanism(s) responsible for its activity have not been fully elucidated. Ding et al. have suggested that CQ may act as a copper ionophore [7, 10]. Alternatively, others have suggested that Cu(CQ)_2_ may act as a proteasome inhibitor, similar to the postulated mechanism of copper diethyldithiocarbamate [12, 13]. CQ and Cu(CQ)_2_ were evaluated in five cancer cell lines of differing origin. Since proteosome inhibitors are part of the treatment regimen in leukemia, the MV-4-411 cell line was selected. Similarly, the use of CQ in Alzheimer’s suggests that it crosses the blood brain barrier and thus the glioma cell line U251 was also tested [5]. As part of a larger initiative, examining the efficacy of copper complexes in platinum resistant cancers, the lung (A549) [14] and ovarian (A2780-S and CP) [15] cell lines were also chosen so as to ascertain the potential of Cu(CQ)_2_ in these cell lines often treated with platinum drugs as first line therapy.

The low aqueous solubility of this copper complex has, however, hindered its development as an anticancer drug candidate. CQ has been tested as a single agent and its use required a mixed solvent system containing DMSO, cremphor, and ethanol [6]. Due to the toxicities associated with such formulations [16], it has not been possible to fully assess the anticancer potential of Cu(CQ)_2_.

The goals of the current study were to (i) develop and characterize a Cu(CQ)_2_ formulation suitable for parenteral administration, (ii) evaluate the efficacy of Cu(CQ)_2_ as an anticancer agent, and (iii) assess the use of Cu(CQ)_2_ as a copper ionophore to boost the anticancer activity ascribed to disulfiram (DSF). We have recently demonstrated that copper complexes can be synthesized inside liposomes [17, 18]. The resultant formulations remain in solution and are suitable intravenous. To our knowledge, the studies presented here are the first to assess the anticancer activity of Cu(CQ)_2_. The formulation could be administered intravenously; however, Cu(CQ)_2_ administered this way did not exert meaningful anticancer activity in vivo, even when used in combination with DSF.

## Materials and methods

### Materials

1,2-Distearoyl-sn-glycero-3-phosphocholine (DSPC) and cholesterol (Chol) were purchased from Avanti Polar Lipids (Alabaster, AL). Sephadex G-50 beads were purchased from GE Healthcare (Chicago, IL). ^3^H-cholesteryl hexadecyl ether (^3^H-CHE) and Pico-Fluor 40 scintillation cocktail were purchased from PerkinElmer Life Sciences (Woodbridge, ON, Canada). Phen Green™ SK, diacetate was obtained from Thermofisher Scientific (Waltham, MA). CQ, copper sulfate (CuSO_4_), HEPES, DSF, and all other chemicals (reagent grade) were purchased from Sigma Aldrich (Oakville, ON, Canada).

### Cell lines

The A549, MV-4-11, and U-251 cell lines were obtained from ATCC. A2780-S and A2780-CP cell lines were obtained from Dr. Mark W. Nachtigal at the University of Manitoba (Winnipeg, Canada). All cell lines were used for up to 18 passages. A2780-S and A2780-CP were maintained in DMEM/F12 (Gibco). MV-4-11, A549, and U251 cells were maintained in IMDM (Gibco), RPMI (Gibco), and DMEM (Gibco), respectively. Media for all cell lines were supplemented with 2-mM L-glutamine (Gibco) and 10% fetal bovine serum (Gibco) and maintained at 37 °C and 5% CO_2_. All cell lines were tested negative for mycoplasma.

### Cytotoxicity assays

For in vitro testing, Cu(CQ)_2_ was synthesized prior to cell treatment by mixing CuSO_4_ and CQ at a 1:2 ratio in DMSO. The final concentration of DMSO that the cells were exposed to was < 0.5%. For combination studies, DSF in DMSO was mixed with CuSO_4_ (in water) or Cu(CQ)_2_ at a 1:1 ratio. Cells lines: A549 (2000 cells/well), A2780-S (1500 cells/well), A2780-CP (1500 cells/well), MV-4-11 (4000 cells/well), and U251 (2500 cells/well) were seeded and grown in 384-well plates for 24 h. They were then treated with CQ or Cu(CQ)_2_ in triplicate wells per concentration for 72 h. Following treatment, cell viability was determined in adherent cell lines (A549, A2780-S, A2780-CP, and U251) via in situ staining with Hoechst 33342 and ethidium homodimer-I to differentiate between viable cells and cells that had lost membrane integrity. These cells were imaged with the INCell Analyzer 2200 (GE Healthcare Life Sciences) and 4 images/well were collected. Viability was assessed in the suspension cell line (MV-4-11) using the Presto Blue™ assay (Life Technologies) following the manufacturer’s instructions. Viability data were normalized to vehicle control (0.5% DMSO in media) and expressed as fraction affected where a value of 1 corresponded to 100% loss of cell viability relative to vehicle controls and 0 which indicated that the treated cells behaved identical to the control cells.

### Flow cytommetry

A2780-CP cells were seeded (250,000 cell/well) in six well plates in DMEM/F12 medium containing 10% FBS and allowed to adhere for 24 h. The cells were treated with copper sulfate (100 μM) or copper CQ (100 μM) for 48 h. The supernatant (to account for floating dead cells) was transferred to 50 mL tubes and combined with adherent cells harvested with 0.25% trypsin EDTA. Cells were washed twice with Hank’s medium without phenol red and pellets were resuspended in Annexin V buffer containing Annexin V-FITC (Annexin V-FITC, Life Technologies/Invitrogen). Samples were incubated on ice for 30 min and then stained with PI at a final concentration of 1 μg/mL. Flow cytommetric analysis was performed with the FACSCalibur flow cytometer (Becton-Dickinson) and acquired date was analyzed with the Cellquest software (Becton-Dickinson). The PI-positive and Annexin V-negative cells were considered necrotic, Annexin V-positive cells (both PI positive and negative cells) were considered apoptotic, and the PI-negative and Annexin V-negative cells were considered viable.

### Phen Green™ FL assay for intracellular copper

Phen Green™ FL was used to assess the amount of copper in A2870-CP cells following incubations in the presence and absence of CQ. Cu reduces the fluorescence intensity of Phen Green™ and thus allows for the identification of Cu entering the cell. Cells were grown to 80–90% confluency and treated with vehicle (0.01% DMSO), CQ (25 μM), CuSO_4_ (100 μM), and CQ/CuSO_4_ (25/100 μM) for 1 h. The cells were washed three times with HBS prior to media replacement with fresh medium containing 5-μM Phen Green™ FL for 0.5 h. Cells were then washed three times with Hanks buffered saline solution and imaged using INCell Analyzer 2200 (excitation 420 nm and emission 538 nm).

### Liposome preparation

The extrusion method for liposome preparation has been well documented by others [19]. Briefly, DSPC and Chol were removed from the freezer and placed in a desiccator for 2 h before being weighed and dissolved in chloroform at a 55:45 mole ratio. A non-exchangeable and non-metabolizable lipid marker ^3^H-CHE was incorporated into the chloroform mixture to achieve a specific activity of approximately 0.025-μCu/mmol total lipid. The solution was dried from chloroform using nitrogen gas and a thin film was generated with further drying under high vacuum for at least 3 h. The lipid film was then rehydrated at 65 °C with unbuffered 300-mM CuSO_4_ (pH 3.5). The resulting multilamellar vesicles underwent 5 freeze (in liquid nitrogen) and thaw (65-°C water bath) cycles [20]. The vesicles were then placed in an extruder (Evonik Transferra Nanosciences, Vancouver) and extruded at 65 °C through stacked 0.1-μm polycarbonate filters at least ten times. The size of the resulting liposomes was determined using quasi-electric light scattering (ZetaPals, Brookhaven). The unencapsulated copper was removed by exchanging the sample into a sucrose (300 mM), HEPES (20 mM), and EDTA (15 mM) buffer (SHE buffer, pH 7.4) by passing the sample through a Sephadex G-50 column equilibrated with the buffer. The resulting solution was then dialyzed against a sucrose (300 mM) and HEPES (20 mM) buffer (SH buffer, pH 7.4) and concentrated using tangential flow to the desired liposomal lipid concentration required for experimental studies. Liposomal lipid concentration was determined by vortexing an aliquot of the liposome solution with scintillation cocktail and measuring ^3^H-CHE by liquid scintillation counting (Packard 1900TR Liquid Scintillation Analyzer).

### Cu(CQ)_2_ synthesis

Copper (CuSO_4_)-containing liposomes in SH buffer (20-mM liposomal lipid) were mixed with CQ powder (5-mg CQ) and then incubated at 40 °C (unless indicated otherwise). Formation of Cu(CQ)_2_ was determined over a 60-min incubation period. Liposome-associated Cu(CQ)_2_ was separated from un-reacted CQ using a Sephadex G-50 column equilibrated with SH buffer. The liposome-containing fractions were analyzed for Cu(CQ)_2_ and lipid concentrations to determine Cu(CQ)_2_ to lipid ratios. Liposomal lipid concentrations were measured by quantifying ^3^H-CHE by liquid scintillation counting as described above and Cu(CQ)_2_ concentrations were determined by dissolving samples in methanol and measuring absorbance at 274 nm using a UV-Vis spectrophotometer.

### Cu(CQ)_2_ dissociation from liposomes

Cu(CQ)_2_-containing liposomes (final liposomal lipid concentration 5 mM) were suspended in SH buffer with 50% (*v*/*v*) FBS and incubated with constant mixing at 37 °C in a water bath. At the indicated time points, an aliquot (100 μL) of the liposome solution was passed through a 1-mL Sephadex G-50 spin column equilibrated with SH buffer. The columns were centrifuged at 680 ×*g* for 3 min at 25 °C. The eluate was assayed for CQ using HPLC and lipid concentration was determined by measuring ^3^H-CHE using scintillation counting. An aliquot (50 μL) of eluate was mixed with 950-μL methanol and the sample was then centrifuged at 10,000 ×*g* for 10 min at 4 °C to pellet precipitated proteins. The supernatant was assayed for CQ using HPLC. In brief, the HPLC assay relied on use of a Waters Alliance HPLC Module 2695 and Empower 2 Software. A 30-μL sample was injected and an isocratic mobile phase of water (pH 3 phosphoric acid) and acetonitrile (60:40) was used at a flow rate of 1 mL/min through a Luna C18 column (5 μm, 4.6 × 150 mm) heated to 55 °C. CQ was detected at 254-nm post-column with a model 996 photodiode array detector (Milford, MA). Pyrrolidine diethyldithiocarbamate was added to samples and standards at an excess of 3-mol equivalents prior to injection to ensure dissociation of CQ from Cu.

### Dose range finding studies with Cu(CQ)_2_

To define a dose of the Cu(CQ)_2_ formulation that was well tolerated, mice (*n* = 3) were given an i.v. injection (lateral tail vein) of Cu(CQ)_2_ using a Monday, Wednesday, and Friday ×2 dosing schedule. These studies also assessed the tolerability of Cu(CQ)_2_ when combined with DSF, where DSF was dosed orally at 100 mg/kg, once daily Monday through Friday for 2 weeks. In these studies, Cu(CQ)_2_ was dosed at 30 mg/kg, Monday, Wednesday, and Friday ×2 weeks. The health status of the animals was monitored following an established standard operating procedure. In particular, signs of ill health were based on body weight loss, change in appetite, and behavioral changes such as altered gait, lethargy, and gross manifestations of stress. When signs of severe toxicity were present, the animals were terminated (isoflurane overdose followed by CO_2_ asphyxiation) for humane reasons. Necropsy was performed to assess other signs of toxicity. The surviving animals were monitored for 2 weeks (14 days) after administration of the last dose of Cu(CQ)_2_ and full necropsies were completed on all treated mice at that time to assess whether there were gross changes in tissue/organ appearance.

### Cu(CQ)_2_ pharmacokinetic studies

Cu(CQ)_2_ was injected i.v. at a dose of 30 mg/kg into CD-1 mice. At selected time points, mice (*n* = 4 per time point) were terminated by isoflurane followed by CO_2_ asphyxiation and blood was collected by cardiac puncture directly into EDTA-coated tubes kept on ice. Blood samples were centrifuged (Beckman Coulter Allegra X-15R) at 1500 ×*g* for 15 min at 4 °C. Plasma was collected and placed into a separate tube prior to assaying for copper, CQ, and liposomal associated lipid. The copper was measured using atomic absorption spectroscopy (AAS) by diluting plasma into 0.1% HNO_3_ and CQ was measured using the HPLC method described above. The amount of liposomal lipid was determined as described above, where plasma (30 μL) was added to Pico-Fluor 40 scintillation cocktail prior to quantifying ^3^H-CHE by liquid scintillation counting.

### Cu(CQ)_2_ efficacy studies in the U251 and A2780-CP subcutaneous tumor models

U-251 cells were grown in culture for four to eight passages prior to inoculation. NRG mice (*n* = 6 per group) were inoculated subcutaneously using a 28-gauge needle into the right flank of the mouse with 5 × 10^6^ cells in a total volume of 50 μL. When the tumors reached 50–100 mm^3^, as measured using digital calipers, animals were given (i.v.) vehicle (SH buffer), CuSO_4_-liposomes (Cu = 3.2 mg/kg), or Cu(CQ)_2_ (CQ = 30 mg/kg, Cu = 3.2 mg/kg) on a Monday, Wednesday, and Friday for 2 weeks. The amount of Cu in Cu(CQ)_2_ liposomes was equivalent to a dose of 3.2 mg/kg and this was the rationale used for dosing of the CuSO_4_-liposome group.

A2780-CP cells were grown in culture for four to eight passages prior to inoculation. NRG mice (*n* = 8 per group) were inoculated subcutaneously using a 28-gauge needle with 1 × 10^6^ cells in a total volume of 50 μL. Treatment was initiated 4 days after cell inoculation and the treatment groups were the same as those indicated above for studies in animals with established U-251 tumors.

Tumor size and body weight were measured three times weekly throughout the study. Animals were terminated by CO_2_ asphyxiation following isoflurane anesthesia when tumors reached a maximum size of 800 mm^3^ or when tumors ulcerated. The health status of the animals was monitored daily following an established standard operating procedure as described above.

### Cu(CQ)_2_ and DSF combination efficacy studies in A2780-CP subcutaneous tumor models

For combination studies, A2780-CP cells were inoculated s.c. in NRG mice (*n* = 13 per group) as outlined above. On day 4, mice were treated i.v. with vehicle (SH buffer), CuSO_4_-liposomes (Cu = 3.2 mg/kg), or Cu(CQ)_2_ (CQ = 30 mg/kg, Cu = 3.2 mg/kg) on a Monday, Wednesday, and Friday ×2-week schedule. Additionally, DSF (100 mg/kg) was dosed by oral gavage Monday through Friday for 2 weeks alone and in combination with the other treatment groups. Tumor size and body weight were measured three times weekly throughout the study. The health status of the animals was monitored daily following an established standard operating procedure as described above. Animals were terminated by CO_2_ asphyxiation following isoflurane anesthesia when tumors reached a maximum size of 800 mm^3^ or when tumors ulcerated.

### Statistical analysis

All data were plotted as mean ± SEM or mean ± SD, as indicated in the figure legends. The IC_50_ of added compounds and 95% confidence intervals (CI) were extrapolated using Prism 6.0 (GraphPad software) from a non-linear regression (curve fit) of the cytotoxicity curves. Statistical analyses comparing tumor growth were performed using one-way ANOVA followed by Tukey adjustments to correct for multiple comparisons. A *P* value < 0.05 was considered statistically significant.

## Results

### Cytotoxicity of CQ and its copper complex

**Fig. 1:**
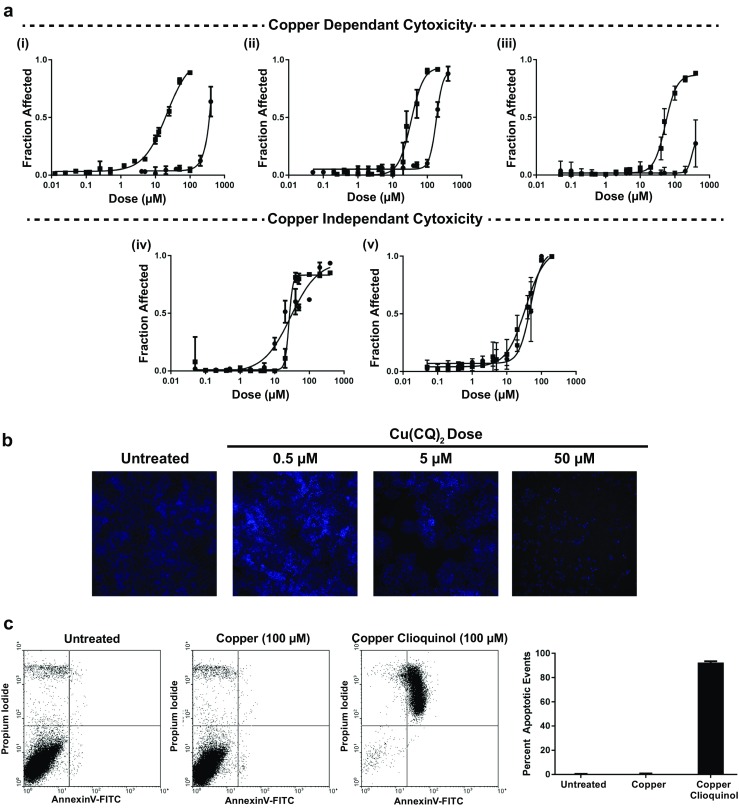
The cytotoxicity of Cu(CQ)_2_ in cancer cell lines. **a** Cytotoxicity curves for CQ (-●-) and Cu(CQ)_2_ (-■-) were obtained for (i) A2780-S, (ii) A2780-CP, (iii) A549, (iv) U251, and (v) MV-4-11 cells. Cells were seeded for 24 h and then treated with CQ or Cu(CQ)_2_ at doses ranging from 0.05–400 μM for 72 h. Cell viability for the adherent cell lines (A2780-S, A2780-CP, A549, U251) was determined using an INCell analyzer 2200, where viability was assessed based on loss of plasma membrane integrity 72 h following treatment, i.e., total cell count and dead cell count were determined using Hoechst 33342 and ethidium homodimer staining, respectively. The viability of the non-adherent cell line (MV-4-11) was measured using PrestoBlue™. Data is presented as mean ± SEM, where the values were determined in triplicate three times. **b** Representative cell micrograph images of untreated or Cu(CQ)_2_ treated (0.5–5 μM) A2780-CP cells stained with Hoechst 33342. **c** A2780-CP cells untreated or treated with copper sulfate (100 μM) or Cu(CQ)_2_ (100 μM) were incubated with Annexin V-FITC and PI. Stained cells were analyzed using flow cytometry, wherein the upper left quadrant shows only PI positive cells, which are necrotic, and lower left quadrant shows viable cells. The lower right quadrant shows Annexin positive cells (early apoptotic) and upper right quadrant shows Annexin and PI positive cells (late apoptosis). The percentage apoptotic events (early and late) were included in a histogram

The activity of CQ and Cu(CQ)_2_ against A2780-S, A2780-CP, A549, U251, and MV-4-11 cells was determined and the results are summarized in Fig. 1. Both compounds were solubilized in a final DMSO concentration of 0.5% and viability was measured 72-h post-treatment. It should be noted that a visual precipitate was observed when CQ or Cu(CQ)_2_ was > 100 μM. The results with A2780-S, A2780-CP, and A549 cells suggest that the activity of CQ is enhanced significantly when added as the copper complex (Fig. 1i–iii). The IC_50_ of Cu(CQ)_2_ was between 20 and 60 μM, while CQ alone showed very little toxicity even at concentrations > 100 μM. In contrast, the activity of CQ and Cu(CQ)_2_ was equivalent when added to U251 and MV-4-11 cell lines (Fig. 1iv and v). The IC_50_ of CQ was 32 and 46 μM, while it was 27 and 32 μM for Cu(CQ)_2_ in U251 and MV-4-11 cells, respectively. Owing to the failure of CQ as a single agent in clinical trial, we focused on the therapeutic potential of Cu(CQ)_2_. In Fig. 1b, representative cell micrograph images of A2780-CP cells treated with Cu(CQ)_2_ are shown. These images show fewer viable cells as the concentration of Cu(CQ)_2_ increases. To further investigate the mechanism of cell death, flow cytommetry studies were completed where A2780-CP cells were stained with Annexin-V and PI following treatment with Cu(CQ)_2_. The results, shown in Fig. 1c, indicate that cells treated with Cu(CQ)_2_ undergo apoptotic cell death. This is consistent with data reported by Schimmer et al. using leukemia cells treated with CQ as a single agent [21].

### Copper CQ liposome characterization

**Fig. 2:**
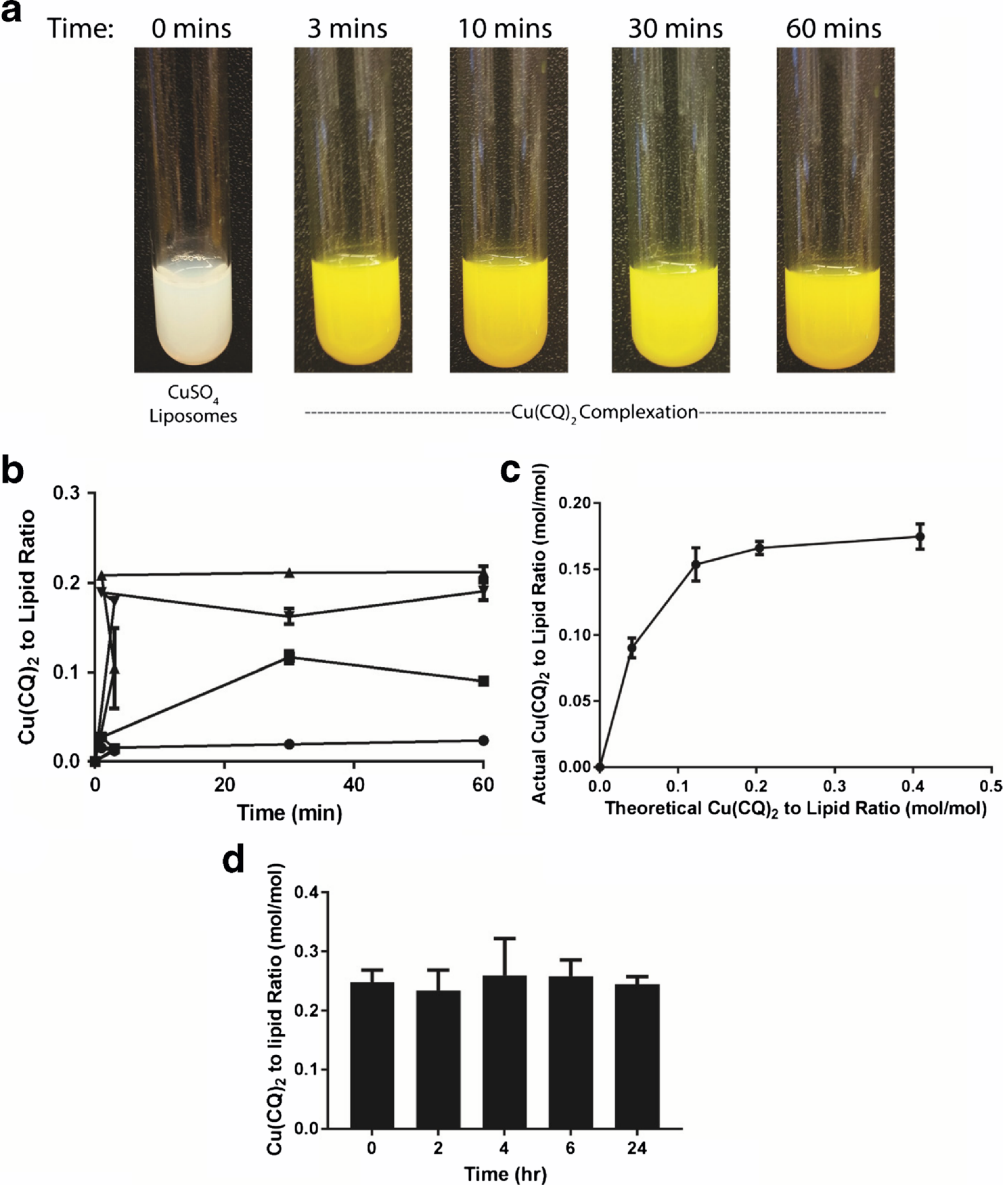
Synthesis of Cu(CQ)_2_ in liposomes prepared with encapsulated 300-mM CuSO_4_. **a** Photograph of solutions consisting of CQ (5 mg/mL) added to CuSO_4_-containing liposomes (20-mM liposomal lipid) over a 1-h time course at 40 °C. **b** Formation of Cu(CQ)_2_ inside DSPC/Chol liposomes (20 mM) as a function of time over 1 h at 4 (●), 25 (■), 40 (▲), and 60 °C (▼) following addition of CQ (5 mg/mL). **c** Measured Cu(CQ)_2_ to liposomal lipid as a function of theoretical Cu(CQ)_2_ to total liposomal lipid ratio estimated based on the amount of CQ added to the liposomes. For these studies, the liposomal lipid concentration was fixed at 20 mM and the added CQ amount was varied. **d** In vitro stability of the Cu(CQ)_2_ formulation over 24 h in 80% fetal bovine serum. Cu(CQ)_2_ was measured using a spectrophotometric assay (**b**–**c**) or HPLC (**d**) and liposomal lipid was measured through use of a radiolabeled lipid (^3^H-CHE). All data are plotted as mean ± SEM

The results summarized above demonstrate that Cu(CQ)_2_ is active against a number of cancer cell lines in vitro, with an IC_50_ ranging from 20 to 60 μM. It is a challenge to develop a drug formulation when the selected drug exhibits activity in the micromolar range and this challenge becomes even greater when the drug is sparingly soluble in aqueous solution. To address this challenge, a formulation method where the copper complex is synthesized inside liposomes was utilized [13]. The data summarized in Fig. 2 demonstrates Cu(CQ)_2_ synthesis inside DSPC/Chol (55:45, mole ratio) liposomes with encapsulated CuSO_4_. The liposomes (20-mM final liposomal lipid concentration) were added directly to 5-mg CQ (as powder) prior to incubating at 40 °C. The color of the solution changed from white to yellow/green within 3 min indicative of Cu(CQ)_2_ formation (Fig. 2a). Formation of Cu(CQ)_2_ was found to be temperature-dependent (Fig. 2b). When samples were incubated at 4 °C, there was no observable color change and the measured Cu(CQ)_2_ to liposomal lipid ratio was less than 0.02 after 60 min. The rate of Cu(CQ)_2_ synthesis was faster as the incubation temperature increased to 25 °C, where the measured Cu(CQ)_2_ to liposomal lipid ratio was 0.1 after 30 min. The optimal temperature for complex formation was 40 °C, where the measured Cu(CQ)_2_ to liposomal lipid ratio was 0.2 after 3 min. In these studies, CQ was added in excess. The amount of Cu(CQ)_2_ formed inside the liposomes will, however, be completely dependent on the amount of copper trapped in the liposome. This is illustrated in Fig. 2c. Increasing the initial theoretical Cu(CQ)_2_ to liposomal lipid ratio beyond 0.15 produced no further increase in the measured Cu(CQ)_2_ to liposomal lipid ratio when using the optimal incubation temperature of 40 °C. In this context, the initial theoretical Cu(CQ)_2_ to liposomal lipid ratio was estimated on the assumption that each mole of copper would complex 2 mol of CQ [11]. When the initial theoretical Cu(CQ)_2_ to liposomal lipid ratio was 0.2, the measured Cu(CQ)_2_ to liposomal lipid ratio was 0.17, which was similar to what was measured for formulations prepared with a large excess of CQ. Preliminary studies assessing the stability of the resulting Cu(CQ)_2_ formulation suggested that less than 10% of the liposome-associated Cu(CQ)_2_ was released from the liposomes when incubated in serum (80%) over 24 h (Fig. 2d).

### Tolerability and pharmacokinetics following i.v. administration of the Cu(CQ)_2_ formulation

**Fig. 3:**
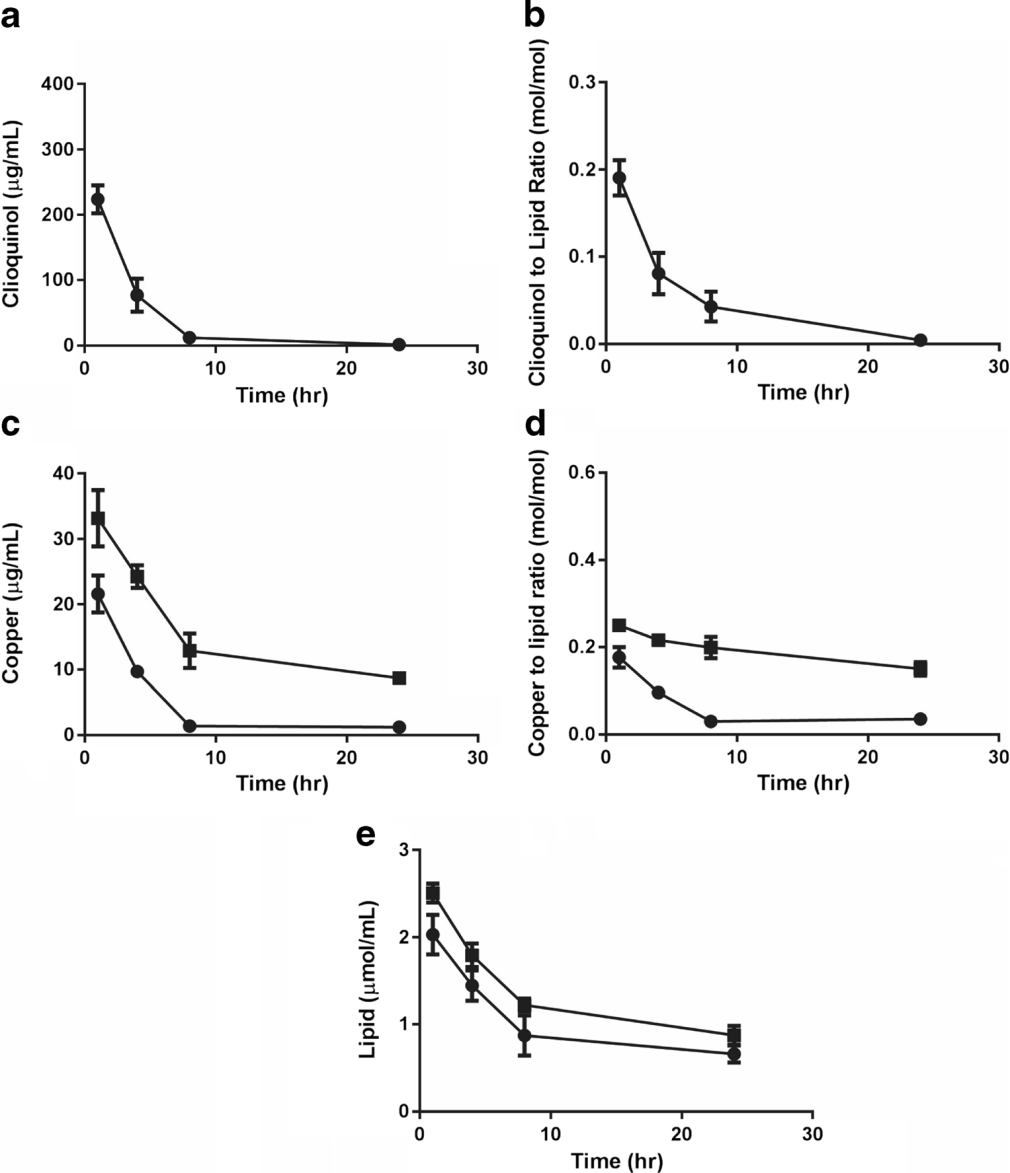
Cu(CQ)_2_ and copper liposome plasma elimination following intravenous injection in CD-1 mice. The Cu(CQ)_2_ liposomes (30-mg/kg CQ, 3.2-mg/kg Cu, 115.6-mg/kg lipid) were dosed in CD-1 mice. Copper liposomes (liposomes prepared in 300-mM CuSO_4_) were injected at the same copper and liposomal lipid dose of 3.2 and 115.6 mg/kg, respectively. **a** CQ plasma concentration over 24 h, where CQ was measured by HPLC methods. **b** CQ to liposomal lipid ratio over 24 h following administration of the Cu(CQ)_2_ formulation. **c** Plasma copper levels following injection of Cu(CQ)_2_ (●) and copper liposomes (■) over 24 h, where Cu^2+^ was measured using AAS (see “Materials and methods”). **d** Copper to liposomal lipid ratio measured over 24 h following injection of copper liposomes or the Cu(CQ)_2_ formulation. **e** The liposomal lipid concentration was measured using scintillation counting of 3H-CHE. All data are plotted as mean ± SEM (*n* = 5), if error bars are not seen they are within the size of the symbol used

The maximum tolerated dose of the Cu(CQ)_2_ formulation was found to be 30 mg/kg when administered i.v. using a dosing schedule of Monday, Wednesday, and Friday for 2 weeks. The formulation was well tolerated at this dose; no weight loss greater than 5% (data not shown) and no notable changes in health status were observed. Following necropsy (14 days after last treatment), there were no gross morphological changes noted. This dose (30 mg/kg) and route of administration was used for the pharmacokinetic studies. The Cu(CQ)_2_ elimination profile was characterized and compared to control liposomes (prepared in 300-mM copper sulfate and exchanged into SH buffer pH 7.4), and the results have been summarized in Fig. 3. For the analysis of plasma samples, an HPLC assay designed to measure CQ was used (see “Materials and methods”) as the measurement of Cu(CQ)_2_ was not possible. At 24-h post-injection of Cu(CQ)_2_, the amount of CQ in the plasma falls below the limit of detection (Fig. 3a). Based on these data, approximately 25% of the injected Cu(CQ)_2_ dose was eliminated within 1 h and greater than 90% was eliminated within 8 h. Assuming the plasma CQ levels measured reflect Cu(CQ)_2_ levels, then it can be estimated that plasma concentrations of Cu(CQ)_2_ are greater than 350, 100, and 20 μM after 1, 4, and 8 h, respectively. It should be noted that this Cu(CQ)_2_ concentration likely represents Cu(CQ)_2_ that is held within liposomes within the plasma compartment and therefore is not representative of the “free” Cu(CQ)_2_ concentration. As illustrated in Fig. 3b, the CQ to liposomal lipid ratio decreases as a function of time after administration. This is indicative of CQ release from the liposomes and into plasma compartment. For example, the measured CQ to liposomal lipid ratio at 4 h is 50% less than that of the injected formulation.

It is not clear from this data that CQ is being released from the liposomes as CQ or Cu(CQ)_2_. For this reason, the plasma copper concentrations were also determined (see “Materials and methods”). The results, shown in Fig. 3c and d, are based on plasma copper levels determined after subtraction of background copper levels determined in plasma obtained from untreated mice. It is assumed, therefore, that the copper being measured is due to the injection of the Cu(CQ)_2_ formulation. As shown in Fig. 3c (filled circles), animals injected with Cu(CQ)_2_ have plasma copper levels that decrease over time, where > 90% of the injected copper dose was eliminated after 8 h. The results shown in Fig. 3d (filled circles) also suggest that the copper to liposomal lipid ratio is decreasing as a function of time after administration. These results were compared to results obtained in animals injected with control liposomes prepared to contain just copper (see “Materials and methods”). This data (filled squares in Fig. 3c and d) indicates that copper elimination is significantly reduced following administration of the copper containing liposomes. This is best illustrated by the results in Fig. 3d, where it appears that the initial copper to liposomal lipid ratio decreases by less than 50% for the copper liposomes but more than 85% for the Cu(CQ)_2_ formulation at 24 h. As noted in Fig. 3e, the elimination of liposomal lipid following administration of Cu(CQ)_2_ and the copper containing liposomes were comparable. In aggregate, the results suggest that following administration of the Cu(CQ)_2_ formulation, both copper and CQ dissociate from the liposomes in the plasma compartment. Since the assays used here were unable to directly measure Cu(CQ)_2_, it was not possible to assess whether the Cu(CQ)_2_ complex is stable following release from the liposomes. It can be concluded that the estimated Cu(CQ)_2_ (assuming CQ is complexed to copper) may be sufficient to engender therapeutic effects based on the IC_50_ of Cu(CQ)_2_ shown above.

### Antitumor efficacy following iv administration of Cu(CQ)_2_

**Fig. 4:**
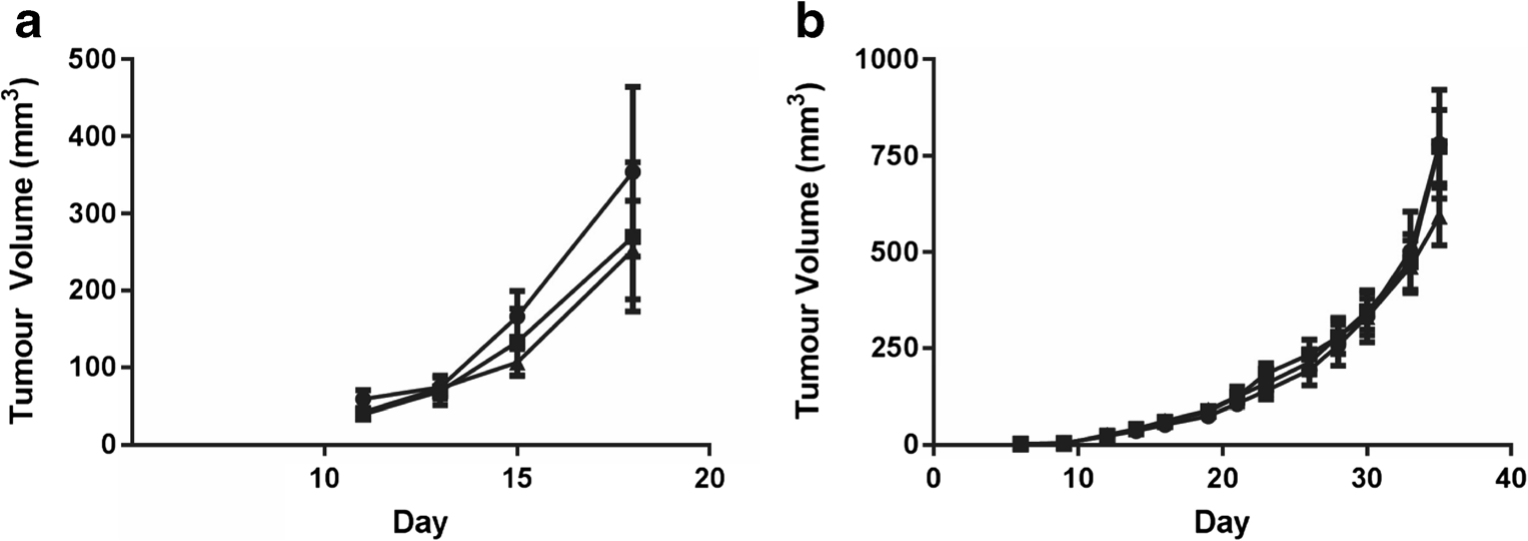
Efficacy of Cu(CQ)_2_ in animals bearing subcutaneous A2780-CP and U251 tumor xenographs. NRG mice with s.c. injected cell lines (see “Materials and methods”) where treatment with vehicle (SH buffer, ●), copper liposomes (liposomes prepared in 300-mM CuSO_4_) (copper dose of 3.2 mg/kg, ■), or Cu(CQ)_2_ (30 mg/kg, ▲). The liposomal lipid dose was 115.6 mg/kg. Treatments were given i.v. on Monday, Wednesday, and Friday for 2 weeks. **a** A2780-CP tumor growth in NRG mice (*n* = 8), dosing began on day 4 post-cell inoculation. **b** U251 tumor growth in NRG mice (*n* = 6), treatment began when tumors reached 50–100 mm^3^. Data is reported as mean ± SEM

An s.c. tumor model of A2780-CP (a platinum-resistant ovarian cancer cell line) was developed; in these cells, the IC_50_ of Cu(CQ)_2_ was approximately 20 μM and the IC_50_ of CQ was > 100 μM. The A2780-CP model is fast growing, where control animals must be terminated due to tumor progression (tumors reach a size > 800 mm^3^, see “Materials and methods”) within 18–22 days following cell injection. For these studies, Cu(CQ)_2_ treatment was initiated 4-days post-cell inoculation. The results, summarized in Fig. 4a, indicate that treatment with copper liposomes and Cu(CQ)_2_ caused a slight, but not significant change in A2780-CP tumor growth rate.

The therapeutic activity of Cu(CQ)_2_ was then evaluated in NRG mice bearing s.c. U251 tumors. This cell line was selected because it was sensitive to both CQ and Cu(CQ)_2_ (Fig. 1); the IC_50_ of Cu(CQ)_2_ and CQ in this glioblastoma cell line was approximately 30 μM. Dosing (30 mg/kg i.v. on Monday, Wednesday, and Friday for 2 weeks) began when the average tumor size reached 50–100 mm^3^.The results, summarized in Fig. 4b, suggest that treatment with Cu(CQ)_2_ had no impact on the growth rate of the U251 tumors when compared to the growth rate in animals treated with the vehicle (SH buffer) or control copper liposomes. Based on these studies, it was concluded that Cu(CQ)_2_, when administered as a single agent, was not efficacious.

### Efficacy of Cu(CQ)_2_ in combination with disulfiram

**Fig. 5:**
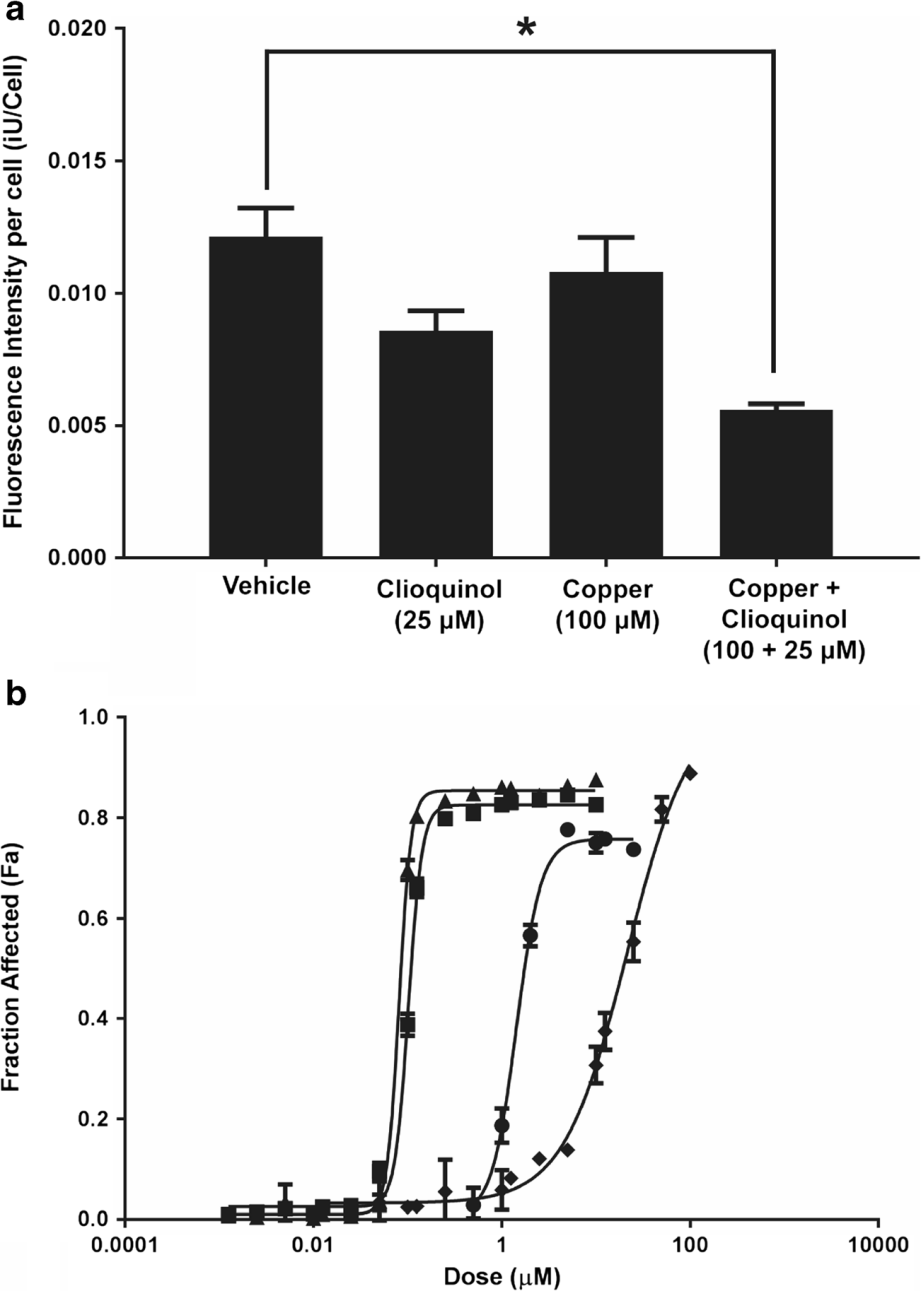
Cu(CQ)_2_-mediated increase in copper delivery to cells and its in vitro activity when combined with disulfiram (DSF). **a** A2780-CP intracellular copper levels were assessed using the cell permeable dye Phen Green™. Cell associated Phen Green™ fluorescence was measured 1-h treatment with the vehicle (0.01% DMSO), CQ, copper, or Cu(CQ)_2_. The cells were then incubated with Phen Green™ for 30 min (see “Materials and methods”). The fluorescence of the probe is quenched in the presence of Cu, and thus a decrease in cell associated fluorescence is indicative of higher intracellular copper levels. Cell-associated fluorescence was measured using an INCell Analyzer 2200. Results shown are an average of three studies done in triplicate (mean ± SEM). **b** Cytotoxicity curves were generated in A2780-CP cells after 72-h treatment with DSF (-●-), Cu(CQ)_2_ (-♦-), or DSF in combination with Cu(CQ)_2_ (-■-), or CuSO_4_ (-▲-). Cell viability was determined using an INCell analyzer 2200, where viability was assessed based on loss of plasma membrane integrity 72 h following treatment, i.e., total cell count and dead cell count were determined using Hoechst 33342 and ethidium homodimer staining, respectively

The studies summarized above suggest that the Cu(CQ)_2_ formulation was not efficacious when administered as a single agent. Cu(CQ)_2_ acts as a copper ionophore [9, 10], thus its potential to act in combination with DSF was explored. The anticancer effects of DSF are dependent on having high intracellular copper levels which could be achieved using a copper ionophore [22]. These studies were completed with the A2780-CP cell line which exhibits sensitivity to Cu(CQ)_2_ but not CQ. The results, summarized in Fig. 3d, are consistent with the published literature, suggesting that Cu(CQ)_2_ can cross lipid bilayers [10]. To illustrate this in a cell model, an assay based on copper-dependent quenching of Phen Green™ fluorescence was used [23, 24]. The results, summarized in Fig. 5, show that the fluorescent intensity of cells incubated with Phen Green™ decreases following addition of Cu(CQ)_2_. This decrease in A2780-CP cell associated Phen Green™ fluorescence was not observed when cells were treated with copper alone. A decrease, albeit not significant, in fluorescence was noted when the cells were treated CQ alone, but this is likely due to CQ-binding copper in the serum-containing cell culture media.

**Fig. 6:**
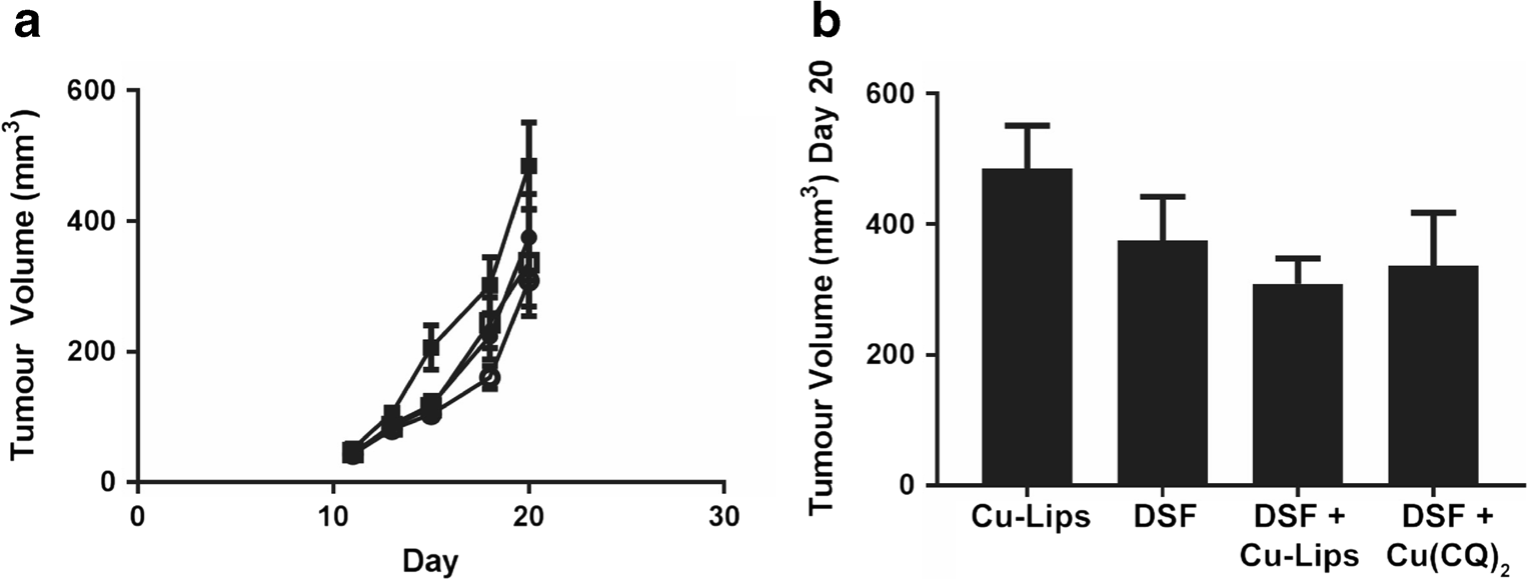
Efficacy of disulfiram (DSF) in combination with Cu(CQ)_2_ and copper liposomes (liposomes prepared in 300-mM CuSO_4_) determined in NRG mice with s.c. A2780-CP tumors. Treatment with CuSO_4_-liposomes (Cu-lips) (copper does of 3.2 mg/kg ■), DSF (100 mg/kg ●), DSF and Cu-liposomes (100-mg/kg DSF and 3.2-mg/kg copper, ○), or DSF and Cu(CQ)_2_ (100-mg/kg DSF and 30-mg/kg Cu(CQ)_2_, □) was initiated 4 days after s.c. inoculation of the A2780-CP cells. Cu(CQ)_2_ and copper liposomes were dosed iv Monday, Wednesday, and Friday for 2 weeks and DSF was dosed orally (see “Materials and methods”) Monday to Friday for 2 weeks. **a** A2780-CP tumor growth in NRG mice (*n* = 13) and **b** tumor size on day 20 was determined as described in the “Materials and methods” data is reported as mean ± SEM

DSF is metabolized to diethyldithiocarbamate (DDC) and DDC can complex with copper to form Cu(DDC)_2_, a cytotoxic agent [17, 25]. To test whether combinations of Cu(CQ)_2_ and DSF were cytotoxic, the agents were added alone and in combination to A2780-CP cells. The results, summarized in Fig. 5b, indicate that cells exposed to Cu(CQ)_2_ (inverted filled triangles) or DSF (filled triangles) alone exhibited compound IC_50_ values of 19 and 1.7 μM, respectively. When DSF was combined with cells with Cu(CQ)_2_ (1:1 ratio), the IC_50_ of DSF decreased to 110 nM. The IC_50_ of DSF in cells treated with Cu(CQ)_2_ is essentially equivalent to the IC_50_ of DSF and CuSO_4_ (90 nM), which indicates that Cu(CQ)_2_ does not inhibit the formation of in vivo activity of Cu(CQ)_2_ when combined with DSF was evaluated in animals bearing s.c. A2780-CP tumors. For these studies, DSF was dosed orally (100 mg/kg) as described elsewhere [26]. DSF-treated animals were dosed concurrently with the Cu(CQ)_2_ formulation or copper liposomes (liposomes prepared in 300-mM CuSO_4_). The results, summarized in Fig. 6, suggest that combinations of DSF with Cu(CQ)_2_-liposomes resulted in a modest, but not significant, reduction in tumor growth rate (Fig. 6a), which could not be differentiated from tumors growing in animals treated with combinations of DSF with copper liposomes. The size of the tumors 20 days after cell inoculation is shown in Fig. 6b.

## Discussion

Recently, efforts have been directed towards repurposing CQ as an anticancer drug [13]. The activity of CQ and Cu(CQ)_2_ against a range of cancer cell lines suggests that Cu(CQ)_2_ is only effective at concentrations ranging from 20 to 60 μM (see Fig. 1). The anticancer activity of CQ alone is worse, with an IC_50_ greater than 100 μM. Interestingly, the activity of CQ is enhanced when administered as a copper complex, although some cell lines show copper independent activity. This may be a consequence of the cellular context in which CQ is presented [3, 27], or it may be due to higher intracellular copper levels in some cell lines when compared to others [21]. Regardless, the preclinical data suggesting CQ anticancer effects was compelling enough to foster initiation of a clinical trial where CQ was given to 11 patients with hematologic malignancies [17]. This study was designed to test whether the metal ionophore activity of CQ and its associated inhibition of the proteasome could engender therapeutic effects in patients with refractory hematologic malignancies. CQ was given orally in a classic dose escalation phase 1 study. The maximum tolerated dose was determined; however, there was minimal activity and no evidence of proteasome inhibition. These authors concluded that the poor activity was due to poor intracellular delivery of CQ [13].

CQ can be administered orally but does suffer from extensive first pass metabolism [2]. This did not affect its utility as an antimicrobial drug but did pose a challenge when attempting to repurpose this drug for cancer, where high plasma concentration is required. One method to overcome first pass metabolism is through i.v. injection. The formulation challenges for both CQ and Cu(CQ)_2_, which are sparingly soluble in water, meant that i.v. dosing was not possible. Herein, a novel formulation of Cu(CQ)_2_ was investigated, where the Cu(CQ)_2_ complex was synthesized inside the core of liposomes suitable for development as a pharmaceutical.

The aqueous core of the liposome is used to carry out a synthesis reaction between copper and CQ; the complex is left in solution (suspended inside the liposome). The amount of Cu inside the liposome is the limiting reagent when forming Cu(CQ)_2_ (see Fig. 2) and the complex formed inside the liposome showed no release in vitro over a time course of 24 h. The formulation appears stable with respect to particle size, polydispersity, and Cu(CQ)_2_ to liposomal lipid ratio. Pharmacokinetic studies completed with the resultant Cu(CQ)_2_ formulation (dosed at 30 mg/kg) indicate that blood levels can be maintained at concentration well above the Cu(CQ)_2_ IC_50_ for at least 8 h after i.v. administration (see Fig. 3). Analysis of the plasma samples strongly suggest that Cu(CQ)_2_ dissociates from the Cu(CQ)_2_ formulation following administration (see Fig. 3d); however, because we did not have an ability to measure Cu(CQ)_2_ in plasma, it was unclear whether the Cu(CQ)_2_ released from the liposomes remained in a complexed form. We believe that this formulation approach addresses the limitation encountered by investigators interested in evaluating CQ activity in patients.

Having overcome the formulation challenges of Cu(CQ)_2_, it was reasonable to ask whether the resulting formulation was efficacious in vivo. Our results suggest that the Cu(CQ)_2_ formulation is not effective, even when administered in combination with DSF, an agent that is significant more potent when combined with a copper [17, 28]. The studies with Cu(CQ)_2_ alone were completed in two subcutaneous tumor models (A2780-CP and U251), representing cell lines in which CQ toxicity was copper-dependent (A2780-CP) and copper-independent (U251). These studies used a dose intensive schedule (Monday, Wednesday, and Friday ×2 weeks) because Cu(CQ)_2_ is active only when present at μM levels. Despite a multidosing schedule and evidence to suggest that the CQ levels in the plasma compartment were above 20 μM for at least 8 h, the Cu(CQ)_2_ formulation did not show any activity. The original studies with CQ were based on its potential to act as a copper ionophore [10], and for this reason, combination studies with DSF were explored. The in vitro results (see Fig. 5) support the fact that DSF/Cu(CQ)_2_ combinations are effective and that nanomolar levels of DSF (in the presence of Cu(CQ)_2_) are sufficient to exert significant cytotoxicity. However, the activity of the combination in vivo (see Fig. 6) indicated otherwise. It can be suggested that the activity of this combination will require an approach that can coordinate the pharmacokinetics of both DSF and Cu(CQ)_2_ such that the two agents reach the tumor site at sufficient levels to achieve effective therapy. It is also possible that sequential, rather than concurrent, dosing may prove beneficial; as was emphasized in studies complete by Verreault et al. [29]. Alternatively, studies have suggested that another 8-hydroxyquinoline analogue, 5-nitro-8-hydroxyquinoline, is much more potent than CQ [18] and future studies could investigate formulations of this analogue. The methodology described here is broadly applicable to the synthesis of many different metal complexes inside liposomes and provides the opportunity to select for formulations that will be better suited for clinical development than the Cu(CQ)_2_ formulation described here.

## Conclusion

This work examined whether CQ and Cu(CQ)_2_ could be formulated in a manner suitable for development as an anticancer agent. A liposomal Cu(CQ)_2_ formulation was described that solves the solubility issues plaguing efforts to assess the activity of the highly water insoluble Cu(CQ)_2_ complex. Further, the resultant formulation ensured that therapeutically effective concentrations of CQ or Cu(CQ)_2_ could be maintained in the plasma compartment over time. However, the resulting formulation was not efficacious whether used alone or in combination with DSF, a drug that is known to be activated in the presence of copper. While this formulation did not exhibit interesting therapeutic effects in vivo, the formulation methods are suitable for other analogues such as 8-hydroxyquinoline which exhibits more potent anticancer effects.
